# Supplementary material for: Acellular Dermal Matrix Used in Diabetic Foot Ulcers: Clinical Outcomes Supported by Biochemical and Histological Analyses
Source: Int J Mol Sci. 2021 Jun 30;22(13):7085. doi: 10.3390/ijms22137085 (PMC8267704; doi:10.3390/ijms22137085)
Supplement: Supplementary file 1 [file ijms-22-07085-s001.zip › ijms-1235502-supplementary.pdf]

Supplementary Material

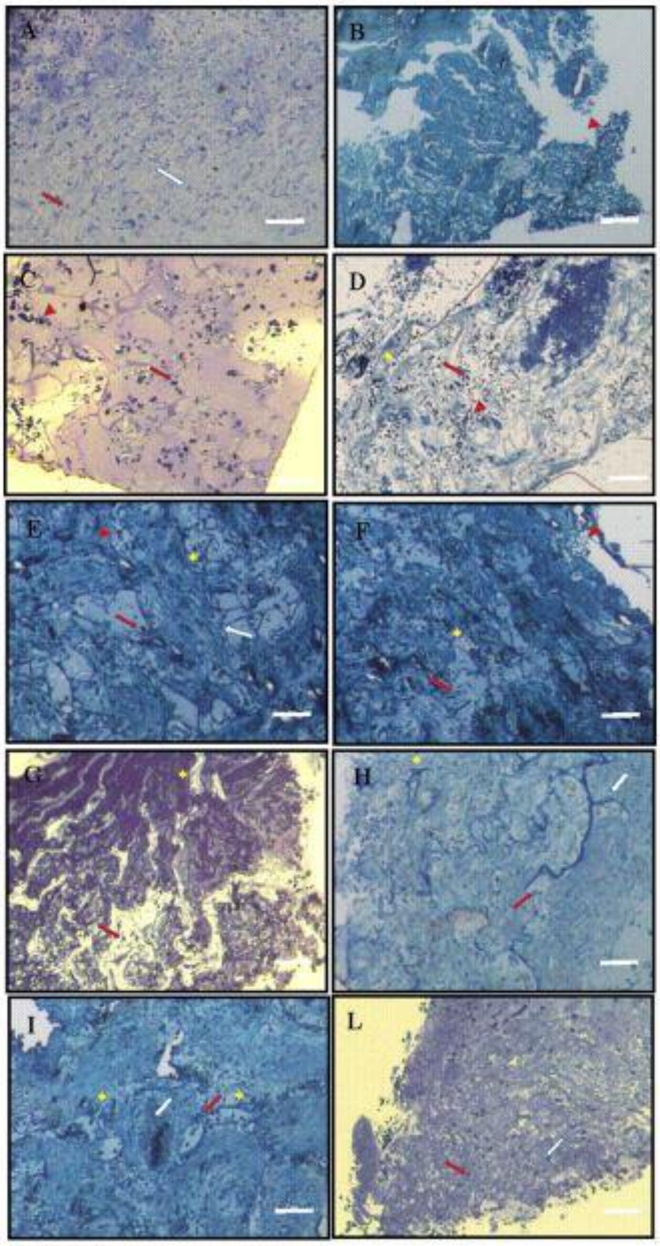

Panel 1

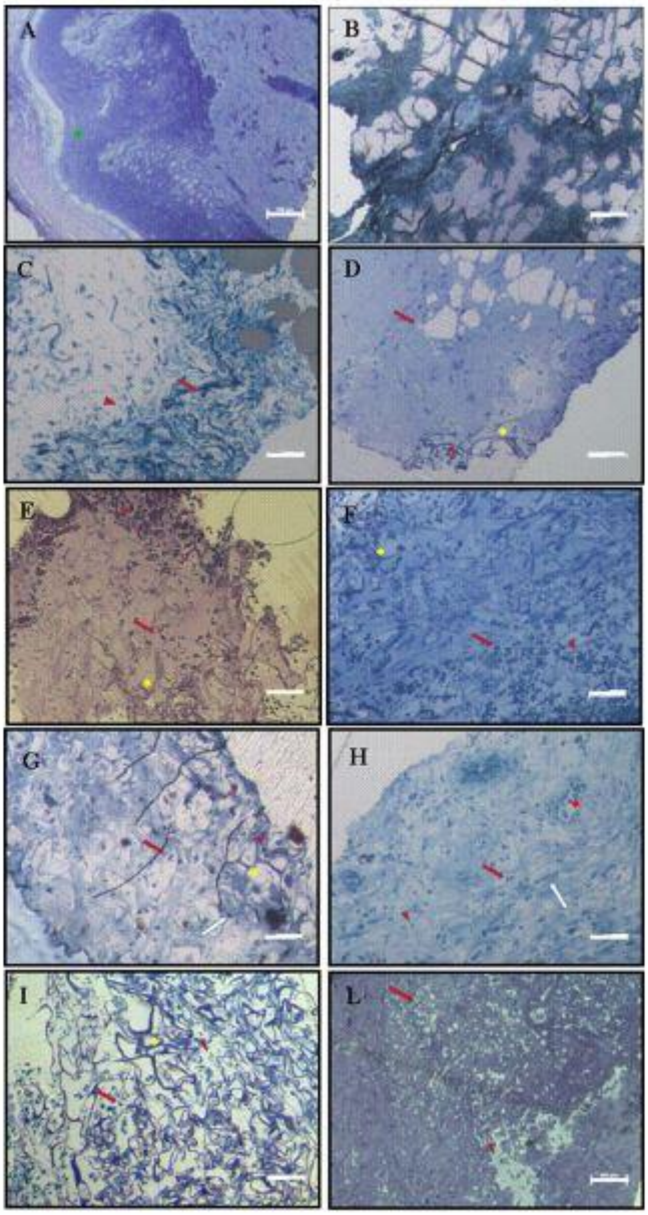

Panel 2

Figure S1: Supplementary material of histological analyses: low magnification histology of tissue biopsies withdrawn at Time 0 and at different post-surgical time points after IDRT or IFWM application. In **Panel I** reports low magnification of histological analyses of patients treated with IDRT. In A and B representative histological images of tissue biopsies withdrawn at time 0 in a healthy part of the skin (A) or in the lesion (B) are reported. From C to L representative microphotographs of the histological appearance of tissue biopsies withdrawn at 7 (C,D), 14 (E,F), 21 (G,H) and 28 (I,L) days after IDRT application are shown. In **Panel II** reports histological analyses of patients treated with IFWM. In A and B representative histological images of tissue biopsies withdrawn at time 0 in a healthy part of the skin (A) or in the lesion (B) are reported. From C to L representative microphotographs of the histological appearance of tissue biopsies withdrawn at 7 (C,D), 14 (E,F), 21 (G,H) and 28 (I,L) days after IFWM application are shown. In both panels the left images (C,E,G,I) represent the lower degree of wound healing that we observed for each time point, whereas the right images (D,F,H,L) represent the higher degree of wound healing that we observed for each time points. Green asterisk indicates epidermis, white arrows indicate fibroblasts; red arrows indicate leukocytes; red arrowhead indicates erythrocyte; yellow asterisk indicates IFWM or IDRT, red asterisks indicate blood vessels. Scale bar = 100 μm

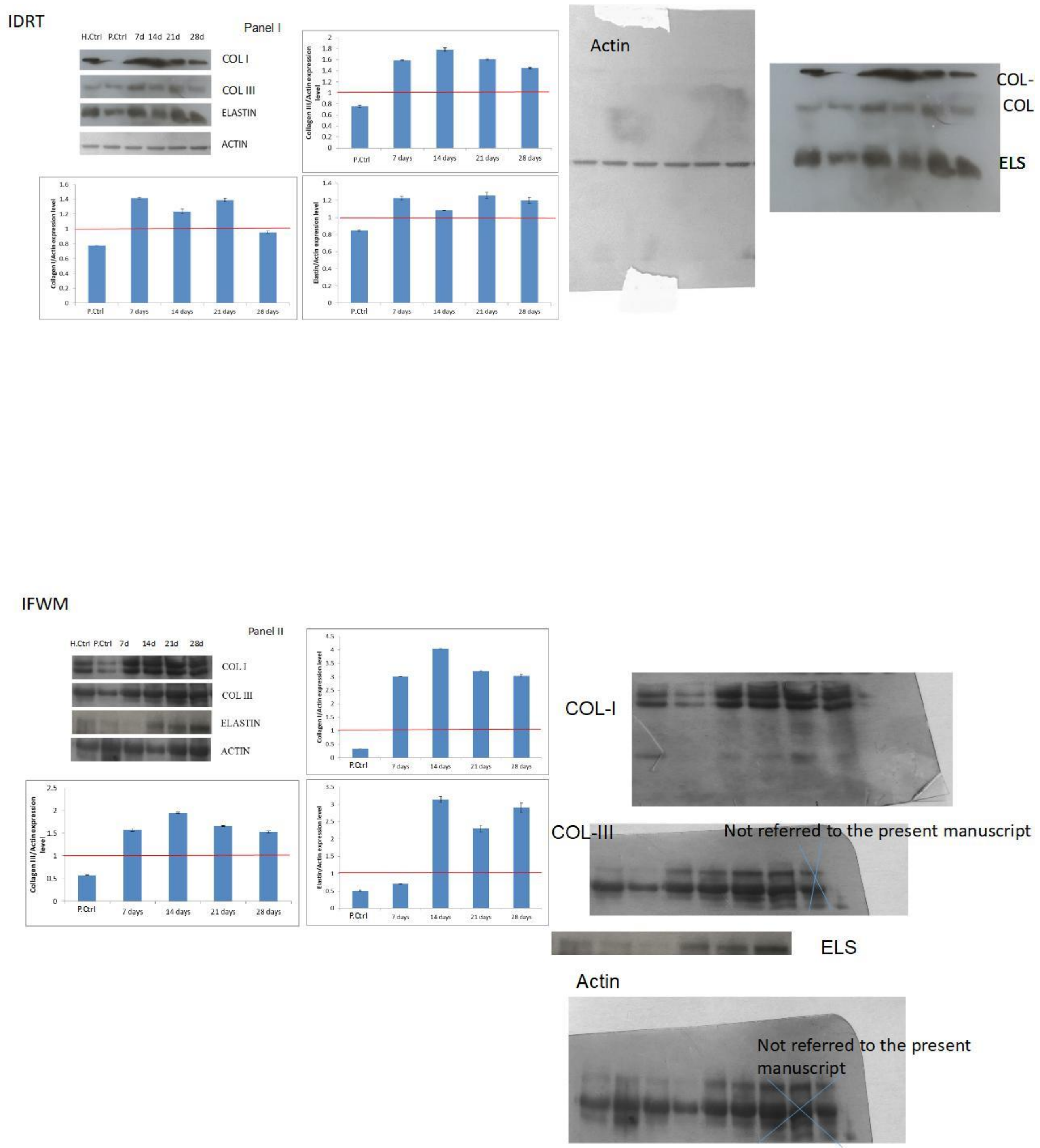

Figure S2: Supplementary materials of western blot analyses, COL-I and III, and elastin protein expression on IDRT- and IFWM-treated lesions. **Panel I**: Western blot analyses on IDRT-treated lesions at 7, 14, 21 and 28 days. H.Ctrl (Healthy Control) represents the sample withdrawn in the healthy part of the skin. P.Ctrl (Pathological Control) represents the sample withdrawn in the lesion before IDRT application. **Panel II**: Western blot analyses on IFWM-treated lesions at 7, 14, 21 and 28 days. H.Ctrl (Healthy Control) represents the sample withdrawn in the healthy part of the skin. P.Ctrl (Pathological Control) represents the sample withdrawn in the lesion before IFWM injection. Next to each panel are provided the original sheet scan relating to the expression of the indicated proteins. Samples of the sixth lane of col III and the last three samples in actin sheet in the second panel do not refer to this manuscript, as indicated.
